# Memory-related default–executive coupling across the lifespan and associations with changes in cognitive control

**DOI:** 10.1162/IMAG.a.1312

**Published:** 2026-08-06

**Authors:** Eirik G. Blakstad, Markus H. Sneve, Didac Vidal-Pineiro, Kristine B. Walhovd, Anders M. Fjell, Håkon Grydeland

**Affiliations:** Center for Lifespan Changes in Brain and Cognition, Department of Psychology, University of Oslo, Oslo, Norway; Department of Radiology and Nuclear Medicine, University of Oslo, Oslo, Norway

**Keywords:** episodic memory, functional connectivity, lifespan, cognitive control, default–executive coupling

## Abstract

Episodic memory and cognitive control decline in aging. The default–executive coupling hypothesis of aging (DECHA) suggests that a neural correlate of cognitive decline in aging is increased functional connectivity (FC) between lateral prefrontal areas and the default-mode network. Here, in a lifespan sample (n = 552, 6–81 years), we tested FC between the left dorsolateral prefrontal cortex (dLPFC) and the default-mode network (DMN) during the encoding and retrieval phases of an episodic memory fMRI task. We created two age groups based on evidence for episodic memory decline after 30 years: a younger group encompassing childhood, adolescence, and young adulthood (6–29 years) and an aging group (30–81 years). To test whether the dLPFC–DMN FC was associated with changes in cognitive control, we used longitudinal change (up to 10 years) in a Stroop inhibition/switching task linked to the prefrontal cortex. Results showed (i) lower dLPFC and DMN connectivity with age in the younger group and higher connectivity in the aging group, and (ii) this FC was associated with an age-related increase in the inhibition task completion time, particularly in the aging group. However, dLPFC showed similar relationships with other networks, particularly salient attentional and control subnetworks, and despite decline in cognitive control associated with memory performance, memory-related FC between dLPFC and DMN did not.

## 1 Introduction

In aging, while a few cognitive processes such as semantic knowledge are relatively resistant, most cognitive processes deteriorate, including episodic memory and inhibitory control functions (Park & Reuter-Lorenz, 2009). This age-related decline in cognitive processing is accompanied by an age-related shift in structural and functional brain properties (Chadick et al., 2014; Turner & Spreng, 2015). To account for these observations, Spreng and Turner (2019) proposed the default–executive coupling hypothesis of aging (DECHA) (Spreng & Turner, 2019; Turner & Spreng, 2015). DECHA posits that cognitive decline is accompanied by an increased and inflexible coupling between the default-mode network (DMN) and lateral prefrontal regions paralleling decline in cognitive control. How this pattern of default–executive coupling unfolds across the lifespan and relates to episodic memory performance remain unclear, and, importantly, longitudinal data are scarce. Here, we tested the main hypotheses of this DECHA model across the lifespan (ages 6–81 years) using measures of longitudinal changes in cognitive control function over 3.5 years and functional connectivity (FC) during incidental encoding and retrieval of episodic memories.

While DECHA does not directly address development, it is broadly known and agreed that cognitive control increases with maturation (Best & Miller, 2010; Constantinidis & Luna, 2019; Luna et al., 2015) and decreases with aging (Ferguson et al., 2021; Manard et al., 2014; Xia et al., 2022). Thus, it is of interest to investigate hypotheses of aging in relation to development (Walhovd et al., 2020) and to evaluate the specificity of aging hypotheses with younger age contrasts. Specifically, DECHA consists of two central hypotheses. The first hypothesis states that general cognitive decline in aging is accompanied by increased functional coupling between the default-mode network (DMN) and the left dorsolateral prefrontal cortex (dlPFC). The DMN is a large-scale brain network that includes medial temporal lobe regions, involved in episodic memory, mental simulation, and future imagination (Andrews-Hanna et al., 2010; Daselaar et al., 2009; Kim, 2010; Kizilirmak et al., 2023). The dlPFC is involved in prioritizing task-relevant information and likely plays a crucial role in cognitive control (Jiang et al., 2018; Petrides, 2005; Turnbull et al., 2019).

In support of this first hypothesis of DECHA, several cross-sectional studies have shown a pattern of greater dlPFC–DMN coupling in older relative to younger adults during tasks of cognitive control (Dixon et al., 2018; Grady et al., 2016; A. C. Murphy et al., 2020). A foundational study demonstrated that higher FC between the left dlPFC and key nodes of the DMN was associated with worsened goal-oriented planning behavior in adults between 63 and 78 years of age, while the same pattern of FC was not found in younger adults (18–27 years), indicating different levels of cognitive control relating differentially to this pattern of functional connectivity (Turner & Spreng, 2015). However, in the context of episodic memory, only one study has associated FC between the DMN and a network including the dlPFC, and episodic memory performance in adults over 70 years of age (Zhukovsky et al., 2023). This association was derived from resting-state fMRI data within a limited age range and in a clinical sample. Hence, it remains unclear how dlPFC–DMN coupling during episodic memory operations evolves with age in normative aging.

The second main hypothesis of DECHA is that the age-related increase in dlPFC–DMN coupling co-occurs with a decline in cognitive control resources (Spreng & Turner, 2019), a hallmark of cognitive aging (Park & Reuter-Lorenz, 2009). Cognitive control refers to the set of processes necessary for planning and executing goal-directed behaviors and for inhibiting irrelevant or competing responses, which in turn likely impact episodic memory performance (Anderson et al., 2016; Badre & Wagner, 2007; Kim, 2020; Ray et al., 2019). According to DECHA, diminished cognitive control and increased default–executive coupling jointly contributes to broader cognitive decline. This loss of control resources is also thought to be accompanied by a shift toward greater reliance on existing knowledge structures, a process referred to as the *semanticization* of cognition (e.g., Park et al., 2002; Singh-Manoux et al., 2012). Lifespan studies of cognitive control have shown age-related differences cross-sectionally (Ferguson et al., 2021) and longitudinally (Adólfsdóttir et al., 2017), including in a partially overlapping sample (Fjell et al., 2017). However, previous studies have not investigated the link between dlPFC–DMN coupling and longitudinal changes in cognitive control, which is critical in determining whether age-related differences in FC associate with a decline in cognitive control resources.

Cognitive control shows consistent improvement from childhood into early adulthood (Best & Miller, 2010; Constantinidis & Luna, 2019; Luna et al., 2015) and some findings suggest that this may happen in parallel with dlPFC–DMN decoupling. For instance, a longitudinal study of participants between the ages of 10 and 13 years found decreased resting-state FC between the DMN and networks of cognitive control (including the left dlPFC) (Sherman et al., 2014), consistent with increasing functional specialization of brain networks during development (Grayson & Fair, 2017). However, other studies with broader age ranges have reported no association between age and dlPFC–DMN connectivity (Betzel et al., 2014; Breukelaar et al., 2020). These inconcistencies leave open the question of how default–executive coupling may differ in earlier stages of life compared with later aging.

Although much of the existing research on DECHA has focused on cognitive control (Dixon et al., 2018; Grady et al., 2016; A. C. Murphy et al., 2020; Turner & Spreng, 2015), the model is intended to explain broader patterns of cognitive decline in aging through underlying brain network dynamics. Episodic memory is a domain that undergoes marked changes across the lifespan and may also be influenced by default–executive network dynamics. Memory performance improves through childhood and adolescence (Selmeczy & Ghetti, 2025; Shing & Lindenberger, 2011), paralleling structural maturation of the medial temporal lobe and dlPFC (Gogtay et al., 2004; Sowell et al., 2003). Decline in episodic memory has been observed as early as the third decade of life in both cross-sectional and longitudinal studies (Capogna et al., 2022; Salthouse, 2009; Vidal-Piñeiro et al., 2019), although considerable inter-individual variability exists and other studies suggest later onset of decline (Nyberg et al., 2012). While the brain mechanisms underlying this variability are likely complex and multifaceted (Nyberg, 2017; Small, 2001; Tromp et al., 2015), DECHA offers a specific and testable mechanism to help explain these variations in memory performance using a task-based functional imaging approach (Mooraj et al., 2025).

The primary aim of the present study was to test predictions derived from the DECHA framework, which concerns aging-related differences in large-scale network interactions between the dlPFC and the DMN. We contrasted findings in later adulthood with early life to evaluate whether the observed associations were specific to aging. Accordingly, participants were stratified into a younger group (6–29 years) and an older group (30–80 years). The 30-year cutoff was informed by episodic memory performance in the current sample. Of note, in addition to greatly facilitating the interpretation of age-related differences across the lifespan, this stratification was intended to provide a test of age invariance (Koen et al., 2019) rather than to delineate specific developmental stages. Compared with other theories of cognitive aging that emphasize dedifferentiation or compensatory recruitment, DECHA makes explicit predictions about task-related default–executive coupling. These predictions make DECHA well suited for evaluation using task-based functional connectivity obtained during episodic memory processing. Importantly, the main predictions of DECHA have not been studied in a healthy lifespan sample to test whether the pattern of FC is specific to aging in the context of episodic memory or with change in cognitive control.

In the present study, we used fMRI during episodic memory encoding and retrieval to test the dlPFC–DMN FC with age in a lifespan sample, and whether this is associated with changes in cognitive control up to 10 years earlier. We also explored the specificity of dlPFC–DMN FC by comparing the dlPFC–DMN coupling with dlPFC coupling with 16 other functional networks (Schaefer et al., 2018). We operationalized cognitive control using completion time in the D-KEFS Stroop inhibition/switching task (Delis et al., 2001), typically associated with cognitive control abilities (Spreng & Turner, 2019). This Stroop subtest captures age-related longitudinal decline in cognitive control well (Clark et al., 2012; Fjell et al., 2017), and is likely dependent on the dlPFC during interference control (Banich, 2019; Liu et al., 2006; Milham et al., 2003). We hypothesized that (i) dlPFC–DMN FC would be greater with age in the aging subsample and inversely, lesser with age in the younger subsample. (ii) We further hypothesized that longitudinal increase in Stroop completion time would be associated with higher levels of dlPFC–DMN FC. (iii) Finally, we expected that such higher levels of dlPFC–DMN FC would be associated with lower memory performance.

## 2 Methods

### 2.1 Participants

**Fig. 1.**
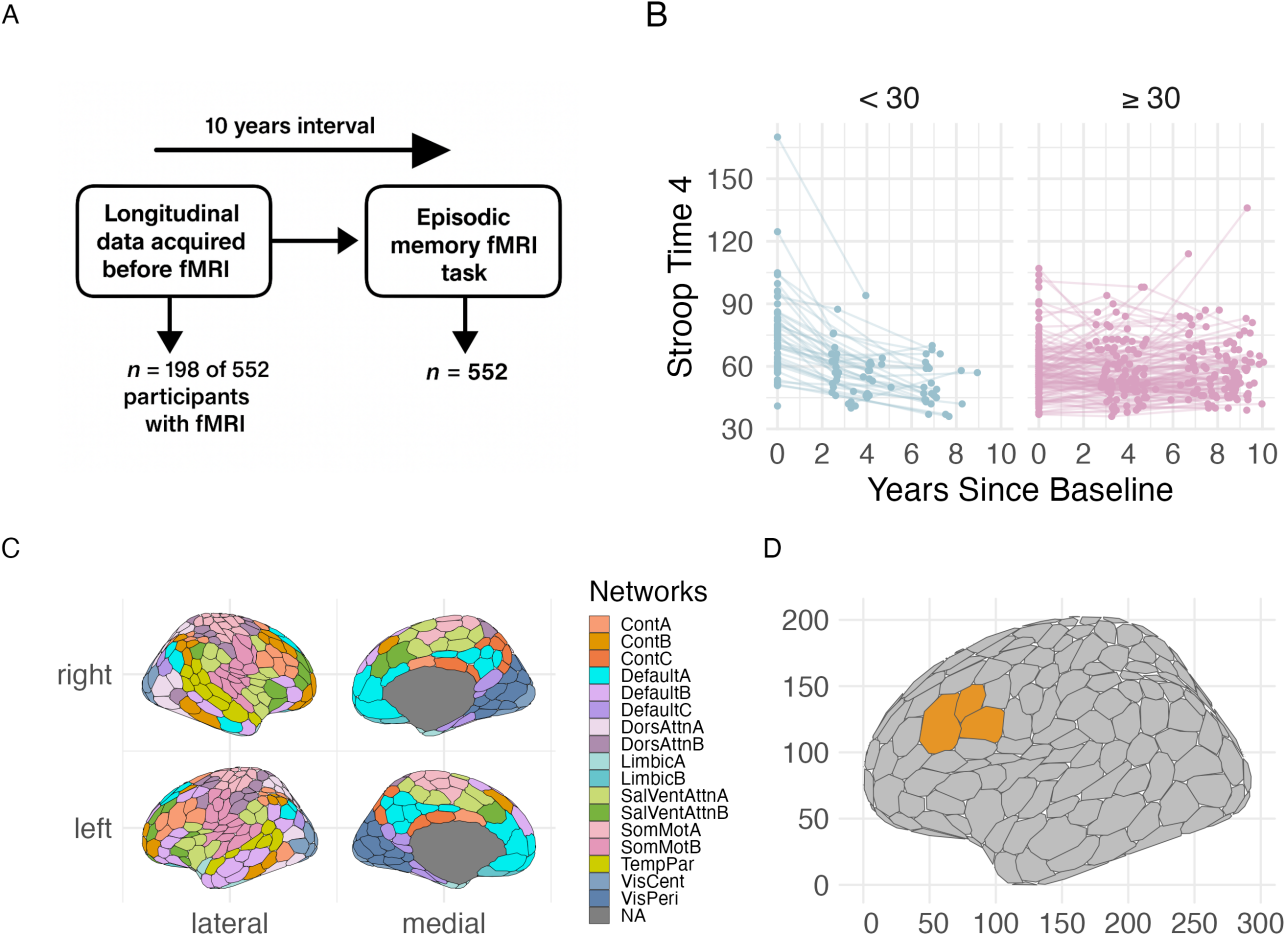
(A) Schematic of study timeline. (B) Descriptive spaghetti plot over participants with multiple Stroop measurements before fMRI acquisition, up to 10 years interval, divided into ages </> 30 years. Points display measurement and lines indicate time between measurements. The last point for each participant is the closest Stroop measurement before fMRI acquisition. (C) Schaefers 17 networks 400 parcellations atlas used to create functional connectivity estimations. (D) Seed regions of left dlPFC found in Control A network from Schaefers 17 networks atlas.

Written informed consent was obtained from all adult participants and from parents or other legal guardians for participants below the age of 18 years. The study was approved by the Regional Ethical Committee of South Norway and conducted in accordance with the Helsinki declaration. A total of 552 healthy participants (371 females, 181 males, mean age = 39.9 years, SD = 18.5, age range = 6–81 years) were part of the cross-sectional fMRI sample (Table 1). Participants who successfully performed the fMRI task underwent screening to ensure they had no history of neurological disorders or chronic illness. They needed to have right-hand dominance and not be taking any medication that may impact the functioning of their nervous system. Additional exclusion of participants was based on neuropsychological criteria including MMSE score for participants > 50 < 26 (Folstein et al., 1975), IQ score < 85 (Wechsler, 1999), and Beck’s Depression Inventory (BDI) > 20 (Beck et al., 1988). A total of 452 of the 552 participants in the cross-sectional sample completed a neuropsychological assessment, including the Stroop task, in connection with the fMRI session. These cognitive assessments were administered as part of the same study wave as the memory fMRI paradigm. Among these 452 participants, 198 had also completed the Stroop task on one or more occasions prior to the fMRI session. The mean (SD) interval between the earliest prior Stroop assessment and fMRI acquisition was 3.5 (2.66) years, with a maximum interval of 10 years (Table 2). Longitudinal analyses were conducted in participants with at least one Stroop assessment prior to fMRI acquisition. In Table 2, “maximum visit number” refers to how many participants attained the visit number as their last before fMRI acquisition. See Figure 1A for a schematic overview of the study design and Figure 1B for the distribution of prior assessments.

**Table 1.** Cross-sectional fMRI sample demographics.

Variables	Mean (SD)	Range	N obs
	Age group < 30		
Age (MRI)	22.39 (6.23)	6 – 29	238
IQ	111.94 (9.47)	89 – 137	154
MMS	29.21 (0.96)	26 – 30	150
BDI	4.74 (3.7)	0 – 14	31
Sex (females/males)			159/79
	Age group ≥ 30		
Age (MRI)	51.30 (14.76)	30 – 81	314
IQ	117.19 (10.19)	90 – 138	212
MMS	29.03 (0.98)	26 – 30	279
BDI	4.23 (3.7)	0 – 16	41
Sex (females/males)			212/102

**Table 2.** Longitudinal sample demographics of participants completing neuropsychological testing up to 10 years before fMRI data collection.

Age group < 30						
Maximum visit number	1 (N = 22)	2 (N = 17)	3 (N = 23)	4 (N = 0)	5 (N = 0)	6 (N = 0)
Age mean (SD)	13.6 (4.08)	16.53 (2.01)	20.19 (4.43)			
Age range	8.89 – 24.60	13.79 – 21.41	15.67 – 29.83			
Stroop mean (SD)	67.8 (12.52)	55.76 (12.48)	52.09 (10.23)			
Stroop range	41 – 90	36 – 94	37 – 70			
Female, N (%)	12 (54.5)	5 (29.4)	12 (52.2)			
Male, N (%)	10 (45.5)	12 (70.6)	11 (47.8)			
Age group ≥ 30						
Max visit number	1 (N = 1)	2 (N = 12)	3 (N = 110)	4 (N = 2)	5 (N = 5)	6 (N = 6)
Age mean (SD)	76.83 (NA)	53.06 (12.07)	60.69 (12.11)	75.78 (7.60)	76.25 (2.70)	75.03 (2.98)
Age range	76.83	33.26 – 68.61	30.93 – 80.89	70.40 – 81.15	71.76 – 78.67	72.10 – 79.97
Stroop mean (SD)	98.00 (NA)	59.58 (15.62)	59.55 (15.17)	61.50 (14.85)	61.20 (16.53)	68.83 (10.17)
Stroop range	98.00	39 – 98	39 – 136	51 – 72	44 – 86	52 – 80
Female, N (%)	0 (0)	4 (33.3)	67 (60.9)	1 (50)	4 (80)	5 (83)
Male, N (%)	1 (NA)	8 (66.7)	43 (39.1)	1 (50)	1 (20)	1 (16.7)

The table summarizes the maximum visit number attained by each participant, defined as the highest completed visit during follow-up. Stroop refers to Stroop completion time in seconds.

### 2.2 Out-of-scanner cognitive measures

For the Stroop color–word interference task, a modified version of the Stroop task was administered (Delis et al., 2001). Out of the four conditions in the task, (1) color naming, (2) reading, (3) inhibition, and (4) inhibition/switching, we used the inhibition/switching condition for our analyses. In this condition, the participants must alternate between reading the colored word and naming the ink color of a word. Specifically, this task measures the response time of naming the color of a word that is either congruent or incongruent with the words’ semantic content and, thereby, the capacity to override the automatic response of reading a word. This design introduces additional demand on cognitive control resources and is shown elsewhere to relate to longitudinal changes (Fjell et al., 2017). The participant is required to complete 48 trials in the condition as fast as possible, where correct and incorrect responses are also noted. To examine age-related changes in semantic knowledge, the Wechsler Abbreviated Scale of Intelligence (WASI) (Wechsler, 1999) vocabulary task was administered. In this task, the participant is read a list of words with increasing difficulty by an examinator, to which they must define the word. We used the raw vocabulary scores to examine age trajectories. Word difficulty is calibrated by age in development (i.e., participants 6–8 years of age start with an easier word than participants 12 years of age).

### 2.3 Experimental design and procedure

The fMRI experiment included an incidental encoding task followed by a retrieval phase after approximately 90 min, both in the scanner. The encoding phase consisted of two runs while the retrieval phase consisted of four runs. All runs started and ended with a 11s baseline recording. Details of the experimental design have been described by others (Sneve et al., 2015; Vidal-Piñeiro et al., 2019). Briefly, during the encoding phase, participants were presented with 100 “everyday objects” in black-and-white drawings. The questions “Can you eat it?” or “Can you lift it?” followed the stimulus presentation. Participants were instructed to respond “yes” or “no” with a button press. The assignment of button presses was counterbalanced across participants. The inter-trial interval ranged from 1 to 7 s and followed an exponential distribution (mean 2.98 s, SD 2.49 s, for additional details, see Sneve et al., 2015). During the retrieval phase, new and old items were presented, preceded by the questions “Have you seen this item before?” If no (new item), another item was presented. If yes (old item), the following question was presented: “Can you remember what to do with the item?” Again, if no, the trial ended. If the participant answered yes, the following question was presented: “Were you supposed to eat it or lift it?” A forced choice associated with the two actions during encoding (eat/lift) was presented and answered with a button press. Classification of responses to old items was (1) source memory (“Yes” response to Question 1 and Question 2, and correct answer to Question 3), (2) item memory (“Yes” response to Question 1 and either “No” to Question 2 or incorrect answer to Question 3), (3) miss (incorrect answer to Question 1). New items were classified either as (4) correct rejections or (5) false alarms. We used (1) source memory as the measure of episodic memory in our analyses.

### 2.4 MRI acquisition

Data were acquired using a 3T MRI (Siemens Skyra Scanner, Siemens Medical Solutions, Germany) and a 20-channel Siemens head–neck coil at Rikshospitalet, Oslo University Hospital. Functional imaging parameters remained consistent throughout all fMRI runs, with a BOLD-sensitive T2*-weighted EPI sequence measuring 43 transversally oriented slices with no gap (TR = 2390 ms, TE = 30 ms, f lip angle = 90◦, voxel size = 3 × 3 × 3 mm^3^, FOV = 224 × 224 mm^2^, interleaved acquisition; generalized autocalibrating partially parallel acquisitions acceleration factor [GRAPPA] = 2). Each encoding run generated 134 volumes, and 3 dummy volumes were acquired at the start of each fMRI run to avoid T1 saturation effects. Anatomical T1-weighted (T1w) magnetization-prepared rapid gradient echo (MPRAGE) images were obtained using a turbo field echo pulse sequence consisting of 176 sagittally oriented slices, while a standard double-echo gradient-echo field map sequence was acquired for distortion correction of the echo planar images. Visual stimuli were presented on a 32-inch LCD monitor, and participants responded using the ResponseGrip device (NordicNeurolab, Norway). Pre-recorded auditory instructions describing the trial structure and task questions were presented via the scanner intercom and delivered through participants’ headphones.

### 2.5 fMRI preprocessing

Data were preprocessed using fMRIPrep (v. 1.5.3; Esteban et al., 2019, 2020) and organized according to the Brain Imaging Dataset Specification (BIDS). The T1-weighted (T1w) anatomical image was corrected for intensity nonuniformity (INU) and used as the reference throughout the pipeline. The T1w-reference was skull stripped, and all functional runs were co-registered to this reference image using boundary-based registration with six degrees of freedom.

Susceptibility distortion correction was performed via fmriprep using standard double echo-fieldmap. Each BOLD run underwent slice-timing correction and motion correction, with motion parameters estimated from rigid body transformations. Denoising was performed using ICA-AROMA (Pruim et al., 2015), incorporating mean white matter and cerebrospinal fluid time series as nuisance regressors, along with the six motion parameters. Detrending and high-pass filtering (0.008 Hz) were applied using a fifth-order Butterworth filter, orthogonalized to the nuisance regressors (Hallquist et al., 2013).

Global signal regression (GSR) was used to reduce global non-neuronal signals that can spuriously inflate functional connectivity estimates (Li et al., 2019; Power et al., 2014). Although GSR can introduce artificial anti-correlations and to some degree remove meaningful neural signals (K. Murphy et al., 2009; Saad et al., 2012), it has also been shown to enhance the specificity of functional networks focused on regionally specific connectivity (Fox et al., 2009; Li et al., 2019). To account for the possibility of results being influenced by preprocessing choice, analyses were also conducted without GSR, in line with recent recommendations by Bijsterbosch et al. (2020).

### 2.6 Functional connectivity estimation

Functional connectivity was estimated with correlational psychophysiological interaction (cPPI) for each individual native space, using an ROI-based approach with partial correlations, controlling for task-unrelated activity (Fornito et al., 2012). The cPPI calculations, including concatenation over runs, were performed using Matlab 2017a, SPM 12.0, and gPPI toolbox 13.1. The cerebral cortex was divided into 400 nodes divided into 17 functional connectivity networks based on the Schaefer et al. (2018) parcellation. To extract cortical time series from the 400-region Schaefer atlas, originally defined in group template (*fsaverage*) surface, we mapped the atlas onto each participant’s native reconstructed surface via FreeSurfer’s *surf2surf* command. In addition, three dlPFC nodes from the ControlA network in the left hemisphere were extracted and were treated as their own network based on ROIs from DECHA (Turner & Spreng, 2015). The cPPI procedure for the same sample during encoding has previously been described (Capogna et al., 2022; Grydeland et al., 2025). Briefly, boxcar functions were used to construct the psychological time series, reflecting 2-s events from the start until the end of each trial. Next, deconvolving BOLD time series from each region the PPI terms were created (Gitelman et al., 2003), and multiplied point-by-point with the psychological time series, followed by re-transforming the PPI terms to BOLD via convolution with a canonical 2-gamma HRF. Finally, cPPI matrices were constructed through partial Pearson’s correlation analysis and the PPI terms are adjusted for the regions’ BOLD time series and the HRF-convolved psychological time series. As described elsewhere (Raud et al., 2023), cPPI estimation of the retrieval phase used all the time points of all three questions to calculate the regressors. However, only the estimates for question 1 were utilized in forming the cPPI matrices. This approach ensures the inclusion of all trials only once, while capturing variability from all task events. The correlation coefficients obtained were Fisher transformed into z-values. Prior to higher-order analyses, average within- and between-network connectivity was computed for the 17 networks and the 3 dlPFC nodes. This was done by averaging the Fisher z-transformed connectivity values between all pairs of ROIs within and across networks, based on the cPPI matrices.

### 2.7 Statistical analysis

#### 2.7.1 Lifespan and longitudinal cognitive trajectories

To characterize and describe age-related patterns in cognitive performance, we used generalized additive models (GAMs) for cross-sectional data and generalized additive mixed models (GAMMs) for longitudinal data, which allow flexible modeling of non-linear and linear trends while accounting for fixed and random effects (Sørensen et al., 2021). Lifespan trajectories of in-scanner episodic memory were modeled using a GAM, while trajectories of cognitive control (Stroop inhibition/switching completion time) and vocabulary performance were modeled using GAMMs.

Longitudinal change in cognitive control and vocabulary was also assessed using separate linear mixed effects models for the younger and aging subsamples. For cognitive control, models included baseline age, sex, Stroop reading, and color naming scores, a test–retest indicator (0 vs. ≥1 previous test), and an interaction between baseline age and time between assessments. Time was modeled as a random slope for each participant. Vocabulary models followed the same structure, excluding Stroop-specific covariates.

#### 2.7.2 Cross-sectional FC–age associations

All continuous predictors were standardized (mean = 0, SD = 1) prior to model fitting, such that regression coefficients reflect standardized effect sizes. To test age-related variation in dlPFC–DMN FC, we used linear regression models with FC as the dependent variable separately for the younger and aging subsamples. Covariates included age, sex, in-scanner movement. Additionally, to isolate region-specific associations, individual mean cortical FC was included as a covariate. This adjustment accounts for global differences in overall connectivity strength across individuals, akin to controlling for total brain volume in regional volumetric analyses. We focused on the DMNa subsystem from the Schaefer et al. (2018) atlas due to its relevance in distractibility and its inclusion of medial prefrontal cortex (mPFC) and posterior cingulate cortex (PCC) nodes (Andrews-Hanna et al., 2010; Turner & Spreng, 2015).

#### 2.7.3 Longitudinal FC–cognitive control associations

To assess whether dlPFC–DMNa FC predicted change in cognitive control, we used GAMMs with Stroop inhibition/switching completion time as the outcome. Models included the interaction between FC and time, along with sex, in-scanner movement, Stroop reading, and color naming scores, and subject-wise average cortical FC, following a level-change modeling approach (Fjell et al., 2023). Analyses were conducted separately for the younger and aging subsamples across all dlPFC–network connections. GAMMs were split by age group to assess age-related contrasts, as non-linear associations observed across the full age range may be driven by only a subset of participants.

#### 2.7.4 Cross-sectional FC–memory associations

We used linear regression models to examine associations between FC and memory performance, including interactions with age. All models included age, sex, in-scanner movement, and subject-wise average cortical FC. Interaction terms were tested in separate models.

#### 2.7.5 Age-stratified analyses

Originally, the sample was stratified into two age segments (6–29 and 30–80 years). Given that the age range 6–29 years likely captures different developmental processes, sensitivity analyses for the main aging models were re-run in a group with participants spanning the more restricted age range of 6–19 years. All statistical models were estimated separately within each segment.

#### 2.7.6 Multiple comparison correction

The associations between dlPFC–DMN functional connectivity and age, Stroop performance change, and memory performance were tested as a priori hypotheses and are, therefore, reported without correction for multiple comparisons. To assess network specificity, all relevant terms (main and interaction) from the models testing dlPFC connectivity with the remaining 16 networks were corrected for multiple comparisons using a 5% false discovery rate (FDR; Benjamini & Hochberg, 1995). All uncorrected main FC findings are given in Supplementary Material (SM), Section 3.

## 3 Results

### 3.1 Age associations for memory, cognitive control, and semantic knowledge

**Fig. 2.**
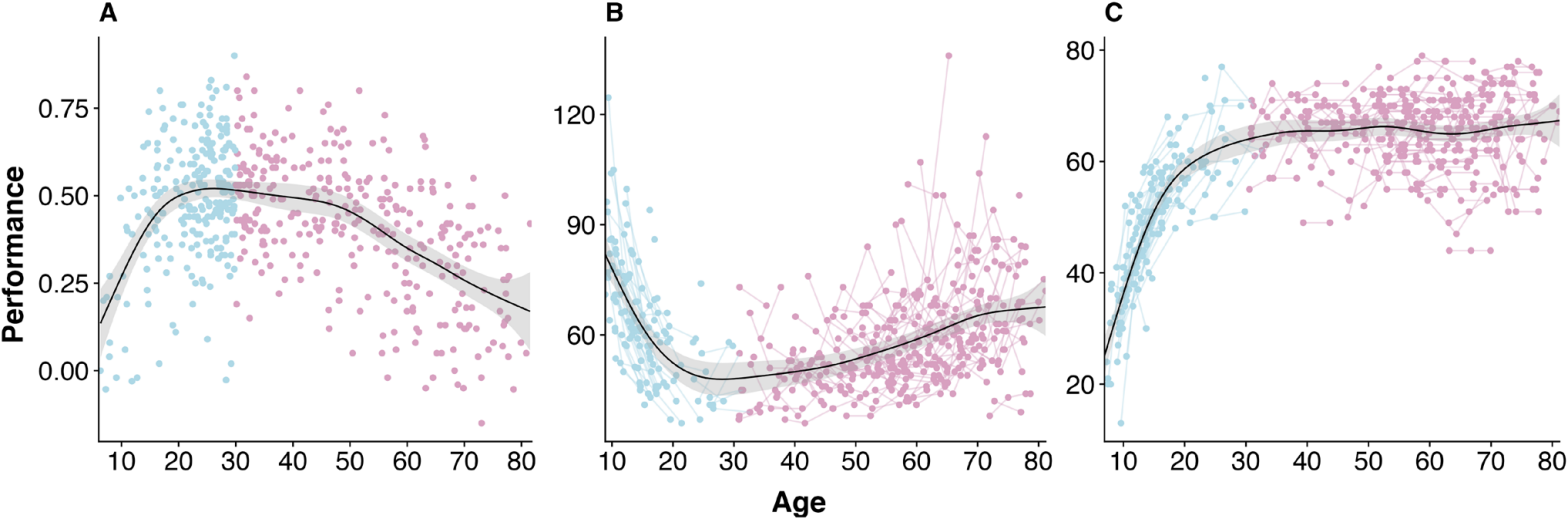
Lifespan trajectories of cognitive measures. (A) Cross-sectional, in-scanner source memory performance, (B) longitudinal, out-of-scanner cognitive control (Stroop inhibition/switching completion time), and (C) vocabulary. Blue indicates age under 30 years and purple indicates the ages over 30 years. Lines between points indicate multiple measurements over time for a participant.

Figure 2A shows the age trajectory of the full lifespan sample of episodic memory performance (*F* = 29.19, edf = 6.73, p < 0.001). The age–memory trajectory showed that performance in the task stabilizes between 20 and 30 years of age, a decline is noticeable from around the 30-year mark, followed by even larger differences starting in the fifth decade of life.

A central hypothesis of DECHA is that the age-related increase in dlPFC–DMNa FC is accompanied by an age-related decline in cognitive control, and a suggested shift toward a greater reliance on prior knowledge, including semantic abilities*.* To investigate this predicted shift in cognition and validate that the data follow these predictions, we used longitudinal measures of cognitive control and semantic abilities taken at the same time as the fMRI and up to 10 years earlier (mean interval = 3.5 years, mean number of measurements per participant = 2.66). Across the full age range, there was a non-linear relationship between age and completion time during the Stroop inhibition condition (Fig. 2B, *F* = 11.54, edf = 5.255, *P* < 0.001). A non-linear relationship with age was also observed for vocabulary (Fig. 2C, *F* = 109.1, edf = 8.307, *P* < 0.001).

For the aging subsample, analyses revealed a positive interaction between age and change, with Stroop completion time change being larger with higher age (SM Fig. 1A, β = 1.62, t = 2.83, p = 0.005). Vocabulary did not show an interaction between age and change (SM Fig. 1B, β = -0.08, t = -0.33, p = 0.7).

As a control analysis (SM Fig. 2), we tested whether changes in Stroop completion time associated with memory performance for the aging subsample. Using GAMM, we found that an increase in Stroop completion time over time was linearly associated with poorer memory performance (edf = 1.00, F. = 6.12 p = 0.01).

### 3.2 Memory functional connectivity and age

#### 3.2.1 dlPFC–DMNa FC and age

**Fig. 3.**
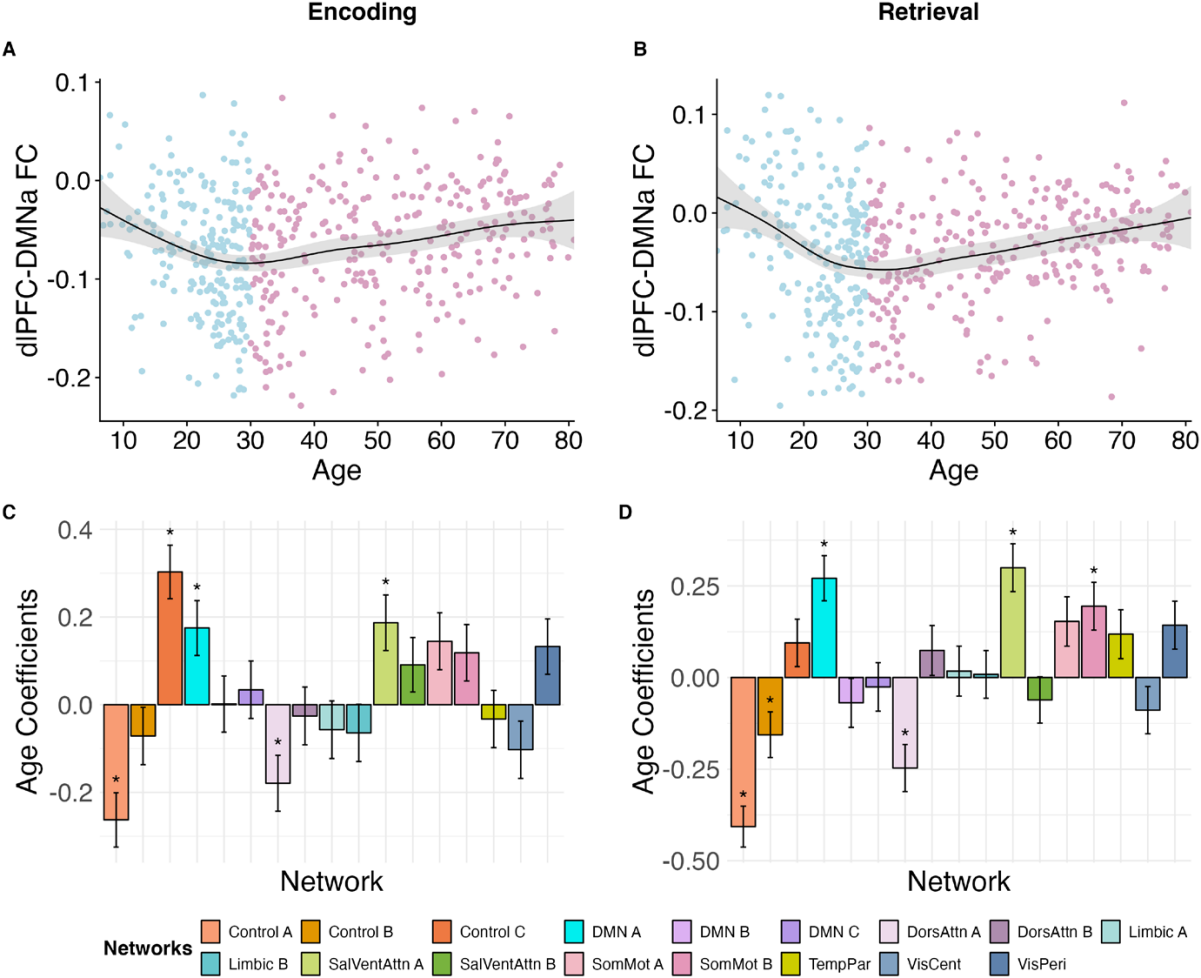
Cross-sectional relationships between age and FC between the dlPFC and DMNa. (A) Lifespan trajectory for dlPFC–DMNa FC during encoding and (B) retrieval. Blue dots represent the younger subgroup, and the purple dots represent the aging subgroup, with the y-axis referring to model residuals adjusting for sex, head movement, and average functional connectivity. (C) All significant age associations for the aging subsample during encoding and (D) during retrieval. The asterisk indicates statistically significant associations after correction for multiple comparisons.

We then examined the main hypothesis of DECHA, namely higher FC between the dlPFC and DMNa with higher age in the aging group, as well as our extension to the younger group, where we expected the inverse relationship. Figure 3A and 3B shows lifespan trajectories during encoding and retrieval, respectively (SM Fig. 3 shows linear age relationships for dlPFC–DMNa FC per age group). Note that values in Figure 3A–B reflect residualized cPPI (Fisher z) values after accounting for mean cortical FC, sex, and in-scanner motion. Raw mean cPPI values for dlPFC–network pairs showing significant age relationships are reported in Supplementary Table S1. In the aging group, linear regression models showed that higher age was associated with higher FC during both encoding β = 0.20, t = 3.2, p = 0.001) and retrieval (β = 0.23, t = 3.66, p < 0.001). In the younger group, higher age was associated with lower FC during retrieval (β = -0.14, t = -1.98, p = 0.04), but not during encoding (β = -0.07, t = -1.09, p = 0.2). This main result was highly similar in both groups when repeating the analyses using FC without performing GSR (SM Fig. 7). In a sensitivity analysis narrowing the age range of the younger group to 6–19 years (n = 59, mean age = 14.10, SD = 3.42), we similarly observed non-significant age coefficient for dlPFC–DMNa during encoding (β = -0.03, t = -0.22, p = 0.82), and a numerically similar, albeit not significant, age coefficient during retrieval (β = -0.16 [vs -0.14 above], t = -0.83, p = 0.40).

#### 3.2.2 Specificity of dlPFC–DMNa FC

To test whether the association between dlPFC and DMNa with age was a specific feature of dlPFC connections, we tested age-related FC between the dlPFC and the remaining 16 networks. As shown in Figure 3C, during encoding for the aging subsample, age was related to higher FC between dlPFC and ControlC (β = 0.30, t = 4.97, FDRp < 0.001), SalventAttnA (β = 0.18, t = 2.95, FDRp = 0.03), and lower FC between dlPFC and ControlA (β = -0.26, t = -4.23, FDRp = 0.001), and DorsAttnA (β = -0.17, t = -2.80, FDRp = 0.01). At retrieval (Fig. 3D), positive relationships with age were observed for SalventAttnA (β = 0.29, t = 4.60, FDRp = 0.001), as well as SomMotB (β = 0.19, t = 2.99, FDRp = 0.01). Negative relationships between FC and age were also observed, between dlPFC and ControlA (β = -0.40, t = -7.29, FDRp < 0.001), DorsAttnA (β = -0.24, t = -3.84, FDRp = 0.002), and ControlB (β = -0.15, t = -2.50, FDRp. = 0.03), respectively. Analyses using FC without GSR demonstrated highly similar age relationships for the networks presented here (SM Fig. 8, ControlC, SalventAttnA, SomMotA-B, and ControlA [retrieval]). However, there were some exceptions: ControlA and DorsAttnA at encoding, as well as ControlB at retrieval, were not significant, and the DorsAttnA network at retrieval showed an age effect in the opposite direction (positive with GSR). Age effects were also seen for SalventAttnB, TempPar, VisCent, VisPeri at encoding, and for DorsAttnB, SomMotA, TempPar, VisCent, VisPeri at retrieval.

For the younger subgroup, no dlPFC–network FC associations with age survived correction for multiple comparisons. Although dlPFC–DMNc FC at encoding did not survive correction for multiple comparisons with GSR, the direction of the slope was consistent with and without GSR. Without GSR, the FC at encoding between dlPFC and DMNc survived FDR correction (β = -0.12, t = -3.09, FDRp = 0.03). In the sensitivity analyses of participants between 6 and 19 years of age (GSR), we observed significant associations for dlPFC–LimbicA FC during encoding (β = 0.49, t. = 3.787, FDRp = 0.007) and retrieval (β = 0.46, t. = 3.07, p = FDRp = 0.03), and dlPFC–DMNc FC during retrieval (β = -0.44, t. = -3.02, FDRp = 0.003) (SM Fig. 4).

### 3.3 Associations between memory FC and preceding change in cognitive control

**Fig. 4.**
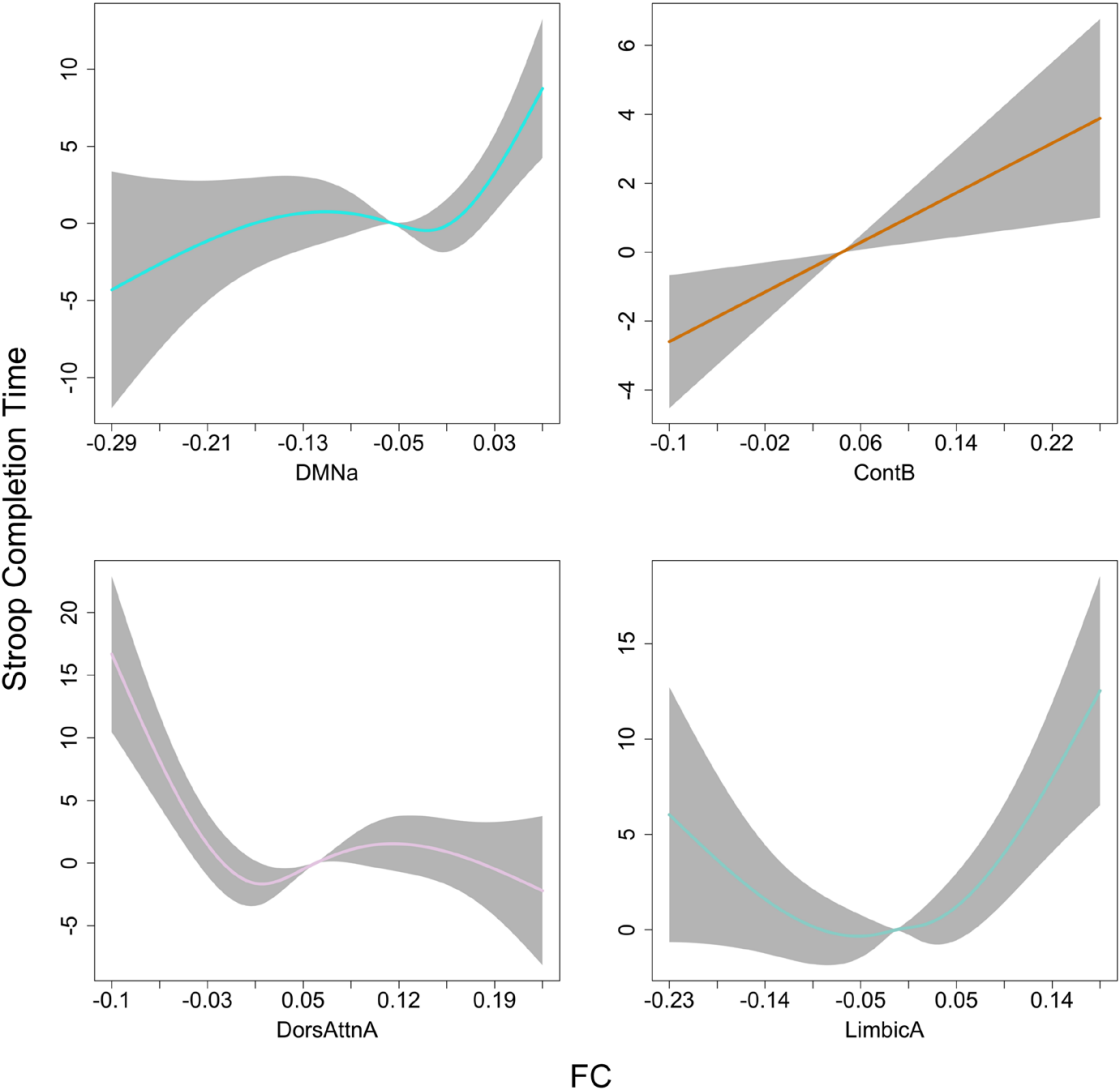
Longitudinal change in cognitive control, as measured by Stroop completion time, varying with dlPFC-to-network FC during encoding. On the y-axis, change in completion time is represented, with positive values indicating an increase in completion time. The x-axis reflects the level of functional connectivity. Upper left panel = DMNa FC during encoding, upper right panel = ControlB FC during encoding, lower left panel = DorsAttna during encoding, lower right panel = LimbicA FC during encoding for the aging subsample.

Using GAMM, we examined whether change in cognitive control related to the level of episodic memory-related FC. As shown in Figure 4 (upper left panel) in the aging group, higher FC between left dlPFC and DMNa related to an increase in Stroop completion time during encoding (edf = 3.26, F = 2.11 p = 0.04), but not during retrieval (edf = 1.00, F = 0.14 p = 0.76). This result was replicated in the FC analysis without GSR (SM Fig. 9).

To test the specificity of dlPFC–DMNa FC relationship with cognitive control change, we tested how dlPFC coupling with other networks associated with cognitive control change. During encoding, increased Stroop completion time correlated with higher FC between left dlPFC and the ControlB network (Fig. 4, upper right panel, edf = 1.00, F = 7.78 p < 0.01, FDRp = 0.04), the LimbicA displayed a U-shape (Fig. 4, lower right panel, edf = 3.17, F = 5.10, p = 0.003, FDRp = 0.01), while decreased Stroop inhibition completion time correlated with higher FC between dlPFC and the DorsAttnA network (Fig. 4, lower left panel, edf = 3.57, F = 8.91 p < 0.001, FDRp = 0.008). Only dlPFC–DorsAttnB FC was significantly associated with longitudinal change in cognitive control during retrieval without GSR (SM Fig. 9). Except for dlPFC–DMNa, no other association was found during encoding after multiple comparison corrections.

Similar analysis in the younger subsample showed a weak, non-significant association for DMNa during retrieval (edf = 2.00, F = 2.35, p = 0.09), but not during encoding (edf =1.00, F = 0.57 p = 0.44).

### 3.4 Relationships with memory performance

Finally, we assessed how task memory performance linked with dlPFC–network FC. In the aging subgroup (SM Fig. 5), memory performance did not associate with dlPFC–DMNa FC at encoding (β = 0.02, t = 0.42, p= 0.66) or at retrieval (β = 0.08, t = -1.55, p. = 0.12). For the other dlPFC couplings, FC between dlPFC and ControlA at retrieval was positively associated with better memory performance after correction for multiple comparisons (β = 0.03, t = 3.16, FDRp = 0.02). For the younger subgroup, we did not observe any associations between FC and memory performance. Similar non-significant results were observed when using FC without GSR.

## 4 Discussion

Our findings are partly consistent with predictions from the DECHA framework. We found that FC between DMN and dlPFC during memory operations was higher in older than in younger adults, and we also observed higher FC in children than in adolescents and young adults. In adults aged 30–81 years, greater dlPFC–DMN FC was associated with longitudinal decline in cognitive control, but not with memory performance. Memory performance instead showed a positive association with connectivity between the dlPFC and nodes within the same control network. Additionally, age-related FC patterns were also observed between the dlPFC and other networks, particularly salient attention and control subnetworks. Taken together, these findings are compatible with key predictions of DECHA, albeit without providing unique evidence in support of this framework. Rather, our results suggest that DECHA at least partly fits a large lifespan framework where broader dlPFC connectivity profiles play prominent roles as neural mechanisms behind cognitive decline in cognitive control and episodic memory.

### 4.1 How does functional connectivity between the default-mode network and the dlPFC varies across the lifespan?

Greater age was related to attenuated anti-correlations between left dlPFC and a DMN subnetwork, partly in line with previous results in adults that have shown both attenuated anti-correlation and higher correlation with age (Grady et al., 2016; Turner & Spreng, 2015; Zhukovsky et al., 2023). The DMNa subnetwork is one of three DMN subnetworks (Yeo et al., 2011) and includes the DMN regions highlighted as important in DECHA, including the medial prefrontal cortex, posterior cingulate cortex, and the angular gyrus (Turner & Spreng, 2015). Our results expand the existing literature by demonstrating a U-shape of dlPFC–DMNa FC across the lifespan. We observed higher levels of FC with higher age in the aging group and lower FC with age in the younger subgroup, specifically during retrieval. However, age-related differences in dlPFC connectivity were not specific to the DMN, as similar age associations were also observed between the dlPFC and other functional networks. Within the DECHA framework, increased FC between the DMN and dlPFC has been interpreted as reflecting a cognitive mode characterized by more mind wandering and distractibility (Spreng & Turner, 2019). This shift is attributed to the putative involvement of the dlPFC in prioritizing neural processing (Petrides, 2005; Jiang et al., 2018; Turnbull et al., 2019) and the DMN in mind-wandering and self-referential associations (Andrews-Hanna et al., 2010, 2014). Thus, according to DECHA, the coupling of these networks predicts a higher degree of distractibility and stronger reliance semanticized knowledge (Spreng & Turner, 2019).

While our results showed an age-related attenuation of the negative dlPFC–DMN correlation, which was associated with reduced cognitive control during encoding, these results are partly consistent with the DECHA framework, but are not uniquely explained by DECHA. Instead, the broader pattern of age-related dlPFC coupling across multiple networks observed here aligns with the growing body of evidence where age-related functional recoupling is characterized by higher FC between prefrontal areas to other regions in the brain with age (Andrews-Hanna et al., 2007; Damoiseaux et al., 2008; Kizilirmak et al., 2023). This pattern has previously also been demonstrated during encoding in the adult part of the sample (Capogna et al., 2022). Specifically, across control networks, we observed different patterns of FC with dlPFC across age in adults. dlPFC nodes originating from Control A were more negatively correlated with Control A with age. Similarly, Control B was more negatively correlated with dlPFC with higher age, whereas dlPFC and Control C correlated more positively with age. Given that control networks are functionally heterogenous (Dixon et al., 2018; Esposito et al., 2018), these opposing associations may reflect differential reorganization of control subsystems with aging, rather than a uniform decline in control connectivity. However, as these findings were included as secondary control analyses, the current data are inconclusive with respect to mechanisms of cognitive control network reorganization in aging.

We found a significant negative age-related slope in the dlPFC–DMN connectivity during retrieval in the younger subsample, whereas encoding did not show a significant age association, but still exhibited a negative age relationship. In the literature, the emerging evidence of dlPFC–DMN coupling in development remains inconclusive. Declining resting-state FC between networks of cognitive control (including the left dlPFC) and the DMN has previously been demonstrated in a longitudinal study between the ages of 10 and 13 years (Sherman et al., 2014), while studies of resting-state FC in longitudinal samples between the ages 8 and 39 years (Breukelaar et al., 2020) and a large cross-sectional lifespan sample (Betzel et al., 2014) did not find correlation between DMN and cognitive control networks FC with age. Our findings indicate that dlPFC–DMN FC evolves throughout the stages of childhood, adolescence, and young adulthood.

The younger subgroup (6–29 years) spans childhood, adolescence, and young adulthood, and this phase should not be interpreted as representing a homogeneous developmental phase. The present study was not designed to delineate fine-grained developmental trajectories or inflection points across this period. Rather, in addition to ease interpretation of lifespan results, the stratification was implemented to contrast later adulthood with early life to evaluate whether the observed associations were specific to older adulthood. Still, re-running analyses with a narrower age group (6–19 years) yielded mainly consistent results as in the 6–29 years age group, although comparisons should be done cautiously due to differences in statistical power.

### 4.2 How does longitudinal changes in cognitive control vary with level of FC?

In aging, we found that dlPFC–DMN FC was positively related to reductions in cognitive control as measured by the Stroop inhibition/switching completion time. This association is consistent with the proposition from DECHA that loss of cognitive control is associated with default–executive coupling in aging (Spreng & Turner, 2019). However, without longitudinal FC measures, this study cannot say whether the changes in cognitive control were accompanied by changes in dlPFC–DMN FC. We did only find a weak (p = 0.09) relationship in the younger group. As we had relatively fewer participants in this group with MRI scans, particularly below 20 years (n = 59), this null finding might be related to statistical power. Still, a challenge when assessing Stroop abilities in children is that reading abilities are still developing that make the Stroop task less effective in capturing “pure” executive functions in children. We addressed this by correcting for color naming in addition to reading abilities in our statistical models, however, this may still be an issue, which in turn may affect how Stroop performance relates to FC in this age group. Hence, we cannot conclude that change in cognitive control only associates with dlPFC–DMNa FC during stages of aging in the lifespan and not to development and early adulthood.

Our results did not point toward specificity of FC from the left dlPFC to the DMNa in relation to loss of cognitive control. We found that left dlPFC connectivity to a network related to cognitive control processing (ControlB) was associated with a longitudinal increase in completion time, potentially demonstrating an increased demand for control processes in aging (Grady, 2012). Moreover, dlPFC connectivity with a network of external attention (DorsAttnA) was related to decreased completion time in the Stroop task. Importantly, this network profile also showed a significant negative age slope. Hence, dlPFC–DMNa and dlPFC–DorsAttnA FC display opposite age relations and opposite relations with cognitive control change. These observations are in line with the well-established anti-correlation between networks of external attention and the DMN (Esposito et al., 2018; Fox et al., 2009). Together, our results suggest that decline in cognitive control is not specifically related to dlPFC–DMN FC but also associate with other dlPFC connections.

Together these findings suggest that declining cognitive control abilities relate to broader lateral–prefrontal interconnectivity beyond just the DMN, potentially reflecting a more general pattern of dedifferentiation where functional networks become less segregated with age (Chan et al., 2014; Grady, 2012). Indeed, we interpret the pattern of attenuated anti-correlation between dlPFC and DMN with age as reflecting neural dedifferentiation, in line with established age-related findings of cognitive control networks and the DMN (Chan et al., 2014). Furthermore, the other FC profiles showing associations with change in cognitive control were all related to networks that show age-related differences, such as, networks of cognitive control, and networks of attention (Dixon et al., 2018; Grady et al., 2016; Rieck et al., 2017). These observations further support the interpretation of these associations relating to neural dedifferentiation. A possible mechanistic interpretation of these results is an age-related reduction in dopaminergic signaling in the caudate and prefrontal cortex that have been linked to less specialized functional connectivity patterns in aging, including evidence for striatal–frontal and striatal–DMN dedifferentiation (Korkki et al., 2025).

### 4.3 Does level of FC relate to memory performance?

While resting-state FC between the dlPFC and DMN has been linked to poor memory performance in older age (Zhukovsky et al., 2023), none of the left dlPFC–network connectivity investigated here was related to memory performance during either encoding or retrieval across the lifespan after correction for multiple comparisons, except for the ControlA network. This discrepancy may be due to differences in sample characteristics, where we used a cognitively healthy lifespan sample, Zhukovsky et al. (2023) associated FC and episodic memory in normally aging participants over 70 years of age and compared this relationship with participants on track to get clinical dementia over 70 years of age. Although our results did not show an association between dlPFC and DMNa FC with memory performance, higher levels of FC between these two regions were observed during both encoding and retrieval with age that indicates that this connectivity profile intrinsically relates to source memory processing.

Although the current study focused on cortical connectivity in relation to memory performance and cognitive control to test specific predictions of the DECHA framework, as well as the specificity of prefrontal–default network associations, cortical–subcortical connections likely also provide important contributions to the inter-individual variation cognitive performance. For instance, cognitive control depends strongly on fronto-striatal circuitry, particularly between dlPFC and the caudate nucleus and the broader striatum (Chatham et al., 2014; Liston et al., 2006; Niendam et al., 2012). This work has demonstrated that dlPFC–striatal coupling plays a central role in executive control, working memory, and goal-directed behavior, and such pathways may, therefore, contribute to longitudinal changes to cognitive control alongside cortical dynamics. Similarly, memory performance is supported by interactions between cortical networks and the hippocampus, where previous research has shown that memory retrieval is enhanced when the DMN is coupled with the hippocampus (Huijbers et al., 2011), and, furthermore, memory performance is strengthened by FC between frontal control networks and DMN when there is a stronger simultaneous hippocampus–DMN connectivity (Westphal et al., 2017). How such cortical–subcortical connectivity interacts and fits into the pattern of dlPFC connection with DMN and other networks constitutes potentially important avenues for future research.

### 4.4 Considerations of functional connectivity estimation and preprocessing

A distinction has been made between context-independent connectivity, reflecting more stable, background coupling, and context-dependent connectivity, reflecting task-evoked modulation beyond baseline connectivity (Cole et al., 2013). In this study, we employed a cPPI analysis to estimate more context-dependent connectivity in the form of task-based FC. cPPI incorporates both intrinsic connectivity and task-evoked modulations by computing the partial correlation between constructed PPI terms of each pair of the cortical regions, controlling background connectivity and task events (Fornito et al., 2012). However, during our work, we have observed that when derived from one task event like source memory compared with baseline, this approach in effect captures context-independent connectivity similar to resting-state FC (Raud et al., 2023). Accordingly, the observed age-related difference in dlPFC–DMN connectivity likely reflects context-independent connectivity differences to a significant extent (Masharipov et al., 2024). Hence, although we do see differences between encoding and retrieval, pointing to task-modulated FC, the age-related differences the dlPFC–DMN coupling observed here might not emerge beyond what would be observed in strictly context-independent FC using residualized background or resting-state FC. Employing alternative connectivity approaches, such as generalized PPI and beta series correlations (McLaren et al., 2012), could to a larger degree probe task-modulated (context-dependent) connectivity.

To assess the reliability of our findings, we examined functional connectivity estimates both with and without global signal regression (GSR). Although GSR remains controversial due to concerns about its potential to introduce artificial anti-correlations and in some cases the removal of meaningful neural variance (K. Murphy et al., 2009; Saad et al., 2012), prior work suggests it enhances network specificity by removing global noise components (Fox et al., 2009; Li et al., 2019; Power et al., 2014). Generally, most associations observed here remained consistent across preprocessing steps, but some networks were significantly and positively associated with age when GSR was not used as a denoising step. This might be due to spurious anti-correlations introduced by GSR (Erdoğan et al., 2016). Furthermore, without GSR, fewer (2 vs 4) dlPFC–network connections related to loss of cognitive control. Still, the main findings of dlPFC–DMNa associations with age and cognitive control were consistently observed across both preprocessing approaches.

### 4.5 Limitations

A main limitation of the current study is the use of cross-sectional functional connectivity and episodic memory data, preventing us from assessing changes over time in these features in relation to the observed longitudinal changes in cognitive control. As participants with more cognitive control decline may already have lower FC at baseline, this lack of longitudinal FC data prevents answering the question of whether decline in cognitive control precedes or co-occurs with FC changes. Hence, future research should investigate simultaneous changes in both FC and cognitive control to determine whether these are level differences already present in FC. Although we used the younger age group as a contrast to determine the specificity of the aging associations, we acknowledge that the age cutoff may not align perfectly with developmental transitions or brain maturation processes occurring during this period. This was amended by adding sensitivity analyses with narrower age range in the younger group. The present analyses were focused on dlPFC–DMN connectivity, with additional dlPFC connectivity to other networks as control analyses to further assess specificity. Although age-related differences in dlPFC connectivity were observed across multiple networks, the analytical approach was based on theoretically motivated connections. This approach precluded determination of the relative contribution of distributed dlPFC network interactions to longitudinal decline in cognitive control. Future studies, using multivariate approaches, such as partial least squares analyses, may provide complementary insights by jointly modeling dlPFC connectivity patterns across networks in relation to longitudinal change in cognitive control. Moreover, in lifespan research, differences in cortical morphology across ages might influence functional measures. Although connectivity was estimated in each participant’s native space, age-related differences in brain morphology may have affected spatial normalization accuracy, However, our use of a surface-based, instead of a volume-based, approach might somewhat have mitigated this issue (Coalson et al., 2018). A limitation of the response-dependent design is variation in trial numbers across participants in the retrieval phase of the in-scanner, which may have led to differences in estimation precision that should be considered when interpreting the results.

## 5 Conclusion

This study showed that dlPFC–DMNa FC varied across the lifespan, with less negative correlation from age 30 years to older adulthood and more negative correlation across childhood, adolescence, and young adulthood. This dlPFC–DMNa pattern was associated with cognitive control decline in older age. Additionally, other dlPFC-to-network FC profiles also exhibited age-related changes and were linked to cognitive control loss, indicating that dlPFC–DMNa coupling is accompanied by other age-related dlPFC couplings that also are associated with cognitive decline. While this coupling was not related to episodic memory performance, it reflects age-related engagement during episodic memory processing. Future research should explore how cognitive control changes relate to longitudinal changes in memory and the coupling between dlPFC and DMN, attentional, and control networks.

## Supplementary Material

Supplementary Material

## Data Availability

The data used in this study are not publicly available due to their sensitive nature and the involvement of human subjects. The code developed for this study is also not publicly available, as it was created for internal use only and is not maintained or documented for external distribution.

## References

[IMAG.a.1312-b1] Adólfsdóttir, S., Wollschlaeger, D., Wehling, E., & Lundervold, A. J. (2017). Inhibition and switching in healthy aging: A longitudinal study. Journal of the International Neuropsychological Society, 23(1), 90–97. 10.1017/S1355617716000898 27938456

[IMAG.a.1312-b2] Anderson, M. C., Bunce, J. G., & Barbas, H. (2016). Prefrontal–hippocampal pathways underlying inhibitory control over memory. Neurobiology of Learning and Memory, 134, 145–161. 10.1016/j.nlm.2015.11.008 26642918 PMC5106245

[IMAG.a.1312-b3] Andrews-Hanna, J. R., Reidler, J. S., Sepulcre, J., Poulin, R., & Buckner, R. L. (2010). Functional-anatomic fractionation of the brain’s default network. Neuron, 65(4), 550–562. 10.1016/j.neuron.2010.02.005 20188659 PMC2848443

[IMAG.a.1312-b4] Andrews-Hanna, J. R., Smallwood, J., & Spreng, R. N. (2014). The default network and self-generated thought: Component processes, dynamic control, and clinical relevance. Annals of the New York Academy of Sciences, 1316(1), 29–52. 10.1111/nyas.12360 24502540 PMC4039623

[IMAG.a.1312-b5] Andrews-Hanna, J. R., Snyder, A. Z., Vincent, J. L., Lustig, C., Head, D., Raichle, M. E., & Buckner, R. L. (2007). Disruption of large-scale brain systems in advanced aging. Neuron, 56(5), 924–935. 10.1016/j.neuron.2007.10.038 18054866 PMC2709284

[IMAG.a.1312-b6] Badre, D., & Wagner, A. D. (2007). Left ventrolateral prefrontal cortex and the cognitive control of memory. Neuropsychologia, 45(13), 2883–2901. 10.1016/j.neuropsychologia.2007.06.015 17675110

[IMAG.a.1312-b7] Banich, M. T. (2019). The stroop effect occurs at multiple points along a cascade of control: Evidence from cognitive neuroscience approaches. Frontiers in Psychology, 10, 2164. 10.3389/fpsyg.2019.02164 31681058 PMC6797819

[IMAG.a.1312-b8] Beck, A. T., Steer, R. A., & Carbin, M. G. (1988). Psychometric properties of the Beck Depression Inventory: Twenty-five years of evaluation. Clinical Psychology Review, 8(1), 77–100. 10.1016/0272-7358(88)90050-5

[IMAG.a.1312-b9] Benjamini, Y., & Hochberg, Y. (1995). Controlling the false discovery rate: A practical and powerful approach to multiple testing. Journal of the Royal Statistical Society Series B: Statistical Methodology, 57(1), 289–300. 10.1111/j.2517-6161.1995.tb02031.x

[IMAG.a.1312-b10] Best, J. R., & Miller, P. H. (2010). A developmental perspective on executive function. Child Development, 81(6), 1641–1660. 10.1111/j.1467-8624.2010.01499.x 21077853 PMC3058827

[IMAG.a.1312-b92] Betzel, R. F., Byrge, L., He, Y., Goñi, J., Zuo, X., & Sporns, O. (2014). Changes in structural and functional connectivity among resting-state networks across the human lifespan. NeuroImage, 102, 345–357. 10.1016/j.neuroimage.2014.07.067 25109530

[IMAG.a.1312-b11] Bijsterbosch, J., Harrison, S. J., Jbabdi, S., Woolrich, M., Beckmann, C., Smith, S., & Duff, E. P. (2020). Challenges and future directions for representations of functional brain organization. Nature Neuroscience, 23(12), 1484–1495. 10.1038/s41593-020-00726-z 33106677

[IMAG.a.1312-b93] Breukelaar, I. A., Griffiths, K. R., Harris, A., Foster, S. L., Williams, L. M., & Korgaonkar, M. S. (2020). Intrinsic functional connectivity of the default mode and cognitive control networks relate to change in behavioral performance over two years. Cortex, 132, 180–190. 10.1016/j.cortex.2020.08.014 32987241

[IMAG.a.1312-b12] Capogna, E., Sneve, M. H., Raud, L., Folvik, L., Ness, H. T., Walhovd, K. B., Fjell, A. M., & Vidal-Piñeiro, D. (2022). Whole-brain connectivity during encoding: Age-related differences and associations with cognitive and brain structural decline. Cerebral Cortex, 33(1), 68–82. 10.1093/cercor/bhac053 35193146 PMC9758575

[IMAG.a.1312-b13] Chadick, J. Z., Zanto, T. P., & Gazzaley, A. (2014). Structural and functional differences in medial prefrontal cortex underlie distractibility and suppression deficits in ageing. Nature Communications, 5(1), 4223. 10.1038/ncomms5223 PMC4088291 24979364

[IMAG.a.1312-b14] Chan, M. Y., Park, D. C., Savalia, N. K., Petersen, S. E., & Wig, G. S. (2014). Decreased segregation of brain systems across the healthy adult lifespan. Proceedings of the National Academy of Sciences, 111(46), E4997–E5006. 10.1073/pnas.1415122111 PMC4246293 25368199

[IMAG.a.1312-b15] Chatham, C. H., Frank, M. J., & Badre, D. (2014). Corticostriatal output gating during selection from working memory. Neuron, 81(4), 930–942. 10.1016/j.neuron.2014.01.002 24559680 PMC3955887

[IMAG.a.1312-b16] Clark, L. R., Schiehser, D. M., Weissberger, G. H., Salmon, D. P., Delis, D. C., & Bondi, M. W. (2012). Specific measures of executive function predict cognitive decline in older adults. Journal of the International Neuropsychological Society, 18(1), 118–127. 10.1017/S1355617711001524 22115028 PMC3314335

[IMAG.a.1312-b17] Coalson, T. S., Van Essen, D. C., & Glasser, M. F. (2018). The impact of traditional neuroimaging methods on the spatial localization of cortical areas. Proceedings of the National Academy of Sciences, 115(27), E6356–E6365. 10.1073/pnas.1801582115 PMC6142239 29925602

[IMAG.a.1312-b18] Cole, M. W., Reynolds, J. R., Power, J. D., Repovs, G., Anticevic, A., & Braver, T. S. (2013). Multi-task connectivity reveals flexible hubs for adaptive task control. Nature Neuroscience, 16(9), 1348–1355. 10.1038/nn.3470 23892552 PMC3758404

[IMAG.a.1312-b19] Constantinidis, C., & Luna, B. (2019). Neural substrates of inhibitory control maturation in adolescence. Trends in Neurosciences, 42(9), 604–616. 10.1016/j.tins.2019.07.004 31443912 PMC6721973

[IMAG.a.1312-b20] Damoiseaux, J. S., Beckmann, C. F., Arigita, E. J. S., Barkhof, F., Scheltens, Ph., Stam, C. J., Smith, S. M., & Rombouts, S. A. R. B. (2008). Reduced resting-state brain activity in the “default network” in normal aging. Cerebral Cortex, 18(8), 1856–1864. 10.1093/cercor/bhm207 18063564

[IMAG.a.1312-b21] Daselaar, S. M., Prince, S. E., Dennis, N. A., Hayes, S. M., Kim, H., & Cabeza, R. (2009). Posterior midline and ventral parietal activity is associated with retrieval success and encoding failure. Frontiers in Human Neuroscience, 3, 13. 10.3389/neuro.09.013.2009 19680466 PMC2726033

[IMAG.a.1312-b22] Delis, D. C., Kaplan, E., & Kramer, J. H. (2001). Delis-Kaplan Executive Function System [Dataset]. In PsycTESTS Dataset. 10.1037/t15082-000

[IMAG.a.1312-b23] Dixon, M. L., De La Vega, A., Mills, C., Andrews-Hanna, J., Spreng, R. N., Cole, M. W., & Christoff, K. (2018). Heterogeneity within the frontoparietal control network and its relationship to the default and dorsal attention networks. Proceedings of the National Academy of Sciences, 115(7), E1598–E1607. 10.1073/pnas.1715766115 PMC5816169 29382744

[IMAG.a.1312-b24] Erdoğan, S. B., Tong, Y., Hocke, L. M., Lindsey, K. P., & deB Frederick, B. (2016). Correcting for blood arrival time in global mean regression enhances functional connectivity analysis of resting state fMRI-BOLD signals. Frontiers in Human Neuroscience, 10, 311. 10.3389/fnhum.2016.00311 27445751 PMC4923135

[IMAG.a.1312-b25] Esposito, R., Cieri, F., Chiacchiaretta, P., Cera, N., Lauriola, M., Di Giannantonio, M., Tartaro, A., & Ferretti, A. (2018). Modifications in resting state functional anticorrelation between default mode network and dorsal attention network: Comparison among young adults, healthy elders and mild cognitive impairment patients. Brain Imaging and Behavior, 12(1), 127–141. 10.1007/s11682-017-9686-y 28176262

[IMAG.a.1312-b26] Esteban, O., Markiewicz, C. J., Blair, R. W., Moodie, C. A., Isik, A. I., Erramuzpe, A., Kent, J. D., Goncalves, M., DuPre, E., Snyder, M., Oya, H., Ghosh, S. S., Wright, J., Durnez, J., Poldrack, R. A., & Gorgolewski, K. J. (2019). fMRIPrep: A robust preprocessing pipeline for functional MRI. Nature Methods, 16(1), 111–116. 10.1038/s41592-018-0235-4 30532080 PMC6319393

[IMAG.a.1312-b27] Esteban, O., Markiewicz, C. J., Goncalves, M., DuPre, E., Kent, J. D., Salo, T., Ciric, R., Pinsard, B., Blair, R. W., Poldrack, R. A., & Gorgolewski, K. J. (2020). *fMRIPrep: A robust preprocessing pipeline for functional MRI* [Computer software]. Zenodo. 10.5281/zenodo.4252786 PMC6319393 30532080

[IMAG.a.1312-b28] Ferguson, H. J., Brunsdon, V. E. A., & Bradford, E. E. F. (2021). The developmental trajectories of executive function from adolescence to old age. Scientific Reports, 11(1), 1382. 10.1038/s41598-020-80866-1 33446798 PMC7809200

[IMAG.a.1312-b29] Fjell, A. M., Sneve, M. H., Grydeland, H., Storsve, A. B., & Walhovd, K. B. (2017). The disconnected brain and executive function decline in aging. Cerebral Cortex (New York, N.Y.: 1991), 27(3), 2303–2317. 10.1093/cercor/bhw082 27073220

[IMAG.a.1312-b30] Fjell, A. M., Sørensen, Ø., Wang, Y., Amlien, I. K., Baaré, W. F. C., Bartrés-Faz, D., Bertram, L., Boraxbekk, C.-J., Brandmaier, A. M., Demuth, I., Drevon, C. A., Ebmeier, K. P., Ghisletta, P., Kievit, R., Kühn, S., Madsen, K. S., Mowinckel, A. M., Nyberg, L., Sexton, C. E., … Walhovd, K. B. (2023). No phenotypic or genotypic evidence for a link between sleep duration and brain atrophy. Nature Human Behaviour, 7(11), 2008–2022. 10.1038/s41562-023-01707-5 PMC10663160 37798367

[IMAG.a.1312-b31] Folstein, M. F., Folstein, S. E., & McHugh, P. R. (1975). “Mini-mental state”: A practical method for grading the cognitive state of patients for the clinician. Journal of Psychiatric Research, 12(3), 189–198. 10.1016/0022-3956(75)90026-6 1202204

[IMAG.a.1312-b32] Fornito, A., Harrison, B. J., Zalesky, A., & Simons, J. S. (2012). Competitive and cooperative dynamics of large-scale brain functional networks supporting recollection. Proceedings of the National Academy of Sciences, 109(31), 12788–12793. 10.1073/pnas.1204185109 PMC3412011 22807481

[IMAG.a.1312-b33] Fox, M. D., Zhang, D., Snyder, A. Z., & Raichle, M. E. (2009). The global signal and observed anticorrelated resting state brain networks. Journal of Neurophysiology, 101(6), 3270–3283. 10.1152/jn.90777.2008 19339462 PMC2694109

[IMAG.a.1312-b34] Gitelman, D. R., Penny, W. D., Ashburner, J., & Friston, K. J. (2003). Modeling regional and psychophysiologic interactions in fMRI: The importance of hemodynamic deconvolution. NeuroImage, 19(1), 200–207. 10.1016/s1053-8119(03)00058-2 12781739

[IMAG.a.1312-b35] Gogtay, N., Giedd, J. N., Lusk, L., Hayashi, K. M., Greenstein, D., Vaituzis, A. C., Nugent, T. F., Herman, D. H., Clasen, L. S., Toga, A. W., Rapoport, J. L., & Thompson, P. M. (2004). Dynamic mapping of human cortical development during childhood through early adulthood. Proceedings of the National Academy of Sciences of the United States of America, 101(21), 8174–8179. 10.1073/pnas.0402680101 15148381 PMC419576

[IMAG.a.1312-b36] Grady, C. (2012). The cognitive neuroscience of ageing. Nature Reviews Neuroscience, 13(7), 491–505. 10.1038/nrn3256 22714020 PMC3800175

[IMAG.a.1312-b37] Grady, C., Sarraf, S., Saverino, C., & Campbell, K. (2016). Age differences in the functional interactions among the default, frontoparietal control, and dorsal attention networks. Neurobiology of Aging, 41, 159–172. 10.1016/j.neurobiolaging.2016.02.020 27103529

[IMAG.a.1312-b38] Grayson, D. S., & Fair, D. A. (2017). Development of large-scale functional networks from birth to adulthood: A guide to neuroimaging literature. NeuroImage, 160, 15–31. 10.1016/j.neuroimage.2017.01.079 28161313 PMC5538933

[IMAG.a.1312-b39] Grydeland, H., Sneve, M. H., Roe, J. M., Raud, L., Ness, H. T., Folvik, L., Amlien, I., Geier, O. M., Sørensen, Ø., Vidal-Piñeiro, D., Walhovd, K. B., & Fjell, A. M. (2025). Network segregation during episodic memory shows age-invariant relations with memory performance from 7 to 82 years. Neurobiology of Aging, 148, 1–15. 10.1016/j.neurobiolaging.2025.01.004 39874716

[IMAG.a.1312-b40] Hallquist, M. N., Hwang, K., & Luna, B. (2013). The nuisance of nuisance regression: Spectral misspecification in a common approach to resting-state fMRI preprocessing reintroduces noise and obscures functional connectivity. NeuroImage, 82, 208–225. 10.1016/j.neuroimage.2013.05.116 23747457 PMC3759585

[IMAG.a.1312-b41] Huijbers, W., Pennartz, C. M. A., Cabeza, R., & Daselaar, S. M. (2011). The hippocampus is coupled with the default network during memory retrieval but not during memory encoding. PLoS One, 6(4), e17463. 10.1371/journal.pone.0017463 21494597 PMC3073934

[IMAG.a.1312-b42] Jiang, J., Wagner, A. D., & Egner, T. (2018). Integrated externally and internally generated task predictions jointly guide cognitive control in prefrontal cortex. eLife, 7, e39497. 10.7554/eLife.39497 30113310 PMC6126922

[IMAG.a.1312-b43] Kim, H. (2010). Dissociating the roles of the default-mode, dorsal, and ventral networks in episodic memory retrieval. NeuroImage, 50(4), 1648–1657. 10.1016/j.neuroimage.2010.01.051 20097295

[IMAG.a.1312-b44] Kim, H. (2020). An integrative model of network activity during episodic memory retrieval and a meta-analysis of fMRI studies on source memory retrieval. Brain Research, 1747, 147049. 10.1016/j.brainres.2020.147049 32781090

[IMAG.a.1312-b45] Kizilirmak, J. M., Soch, J., Schütze, H., Düzel, E., Feldhoff, H., Fischer, L., Knopf, L., Maass, A., Raschick, M., Schult, A., Yakupov, R., Richter, A., & Schott, B. H. (2023). The relationship between resting-state amplitude fluctuations and memory-related deactivations of the default mode network in young and older adults. Human Brain Mapping, 44(9), 3586–3609. 10.1002/hbm.26299 37051727 PMC10203811

[IMAG.a.1312-b46] Koen, J. D., Hauck, N., & Rugg, M. D. (2019). The relationship between age, neural differentiation, and memory performance. The Journal of Neuroscience: The Official Journal of the Society for Neuroscience, 39(1), 149–162. 10.1523/JNEUROSCI.1498-18.2018 30389841 PMC6325265

[IMAG.a.1312-b47] Korkki, S. M., Johansson, J., Nordin, K., Pedersen, R., Bäckman, L., Rieckmann, A., & Salami, A. (2025). Dedifferentiation of caudate functional organization is linked to reduced D1 dopamine receptor availability and poorer memory function in aging. Imaging Neuroscience, 3, imag_a_00462. 10.1162/imag_a_00462 PMC12319749 40800906

[IMAG.a.1312-b48] Li, J., Kong, R., Liégeois, R., Orban, C., Tan, Y., Sun, N., Holmes, A. J., Sabuncu, M. R., Ge, T., & Thomas Yeo, B. T. (2019). Global signal regression strengthens association between resting-state functional connectivity and behavior. NeuroImage, 196, 126–141. 10.1016/j.neuroimage.2019.04.016 30974241 PMC6585462

[IMAG.a.1312-b49] Liston, C., Watts, R., Tottenham, N., Davidson, M. C., Niogi, S., Ulug, A. M., & Casey, B. J. (2006). Frontostriatal microstructure modulates efficient recruitment of cognitive control. Cerebral Cortex, 16(4), 553–560. 10.1093/cercor/bhj003 16033925

[IMAG.a.1312-b50] Liu, X., Banich, M. T., Jacobson, B. L., & Tanabe, J. L. (2006). Functional dissociation of attentional selection within PFC: Response and non-response related aspects of attentional selection as ascertained by fMRI. Cerebral Cortex, 16(6), 827–834. 10.1093/cercor/bhj026 16135781

[IMAG.a.1312-b51] Luna, B., Marek, S., Larsen, B., Tervo-Clemmens, B., & Chahal, R. (2015). An integrative model of the maturation of cognitive control. Annual Review of Neuroscience, 38, 151–170. 10.1146/annurev-neuro-071714-034054 PMC5661874 26154978

[IMAG.a.1312-b52] Manard, M., Carabin, D., Jaspar, M., & Collette, F. (2014). Age-related decline in cognitive control: The role of fluid intelligence and processing speed. BMC Neuroscience, 15(1), 7. 10.1186/1471-2202-15-7 24401034 PMC3890570

[IMAG.a.1312-b53] Masharipov, R., Knyazeva, I., Korotkov, A., Cherednichenko, D., & Kireev, M. (2024). Comparison of whole-brain task-modulated functional connectivity methods for fMRI task connectomics. Communications Biology, 7(1), 1402. 10.1038/s42003-024-07088-3 39462101 PMC11513045

[IMAG.a.1312-b54] McLaren, D. G., Ries, M. L., Xu, G., & Johnson, S. C. (2012). A generalized form of context-dependent psychophysiological interactions (gPPI): A comparison to standard approaches. NeuroImage, 61(4), 1277–1286. 10.1016/j.neuroimage.2012.03.068 22484411 PMC3376181

[IMAG.a.1312-b55] Milham, M. P., Banich, M. T., Claus, E. D., & Cohen, N. J. (2003). Practice-related effects demonstrate complementary roles of anterior cingulate and prefrontal cortices in attentional control. NeuroImage, 18(2), 483–493. 10.1016/S1053-8119(02)00050-2 12595201

[IMAG.a.1312-b56] Mooraj, Z., Salami, A., Campbell, K. L., Dahl, M. J., Kosciessa, J. Q., Nassar, M. R., Werkle-Bergner, M., Craik, F. I. M., Lindenberger, U., Mayr, U., Rajah, M. N., Raz, N., Nyberg, L., & Garrett, D. D. (2025). Toward a functional future for the cognitive neuroscience of human aging. Neuron, 113(1), 154–183. 10.1016/j.neuron.2024.12.008 39788085 PMC13032885

[IMAG.a.1312-b57] Murphy, A. C., Bertolero, M. A., Papadopoulos, L., Lydon-Staley, D. M., & Bassett, D. S. (2020). Multimodal network dynamics underpinning working memory. Nature Communications, 11(1), 3035. 10.1038/s41467-020-15541-0 PMC7295998 32541774

[IMAG.a.1312-b58] Murphy, K., Birn, R. M., Handwerker, D. A., Jones, T. B., & Bandettini, P. A. (2009). The impact of global signal regression on resting state correlations: Are anti-correlated networks introduced? NeuroImage, 44(3), 893–905. 10.1016/j.neuroimage.2008.09.036 18976716 PMC2750906

[IMAG.a.1312-b59] Niendam, T. A., Laird, A. R., Ray, K. L., Dean, Y. M., Glahn, D. C., & Carter, C. S. (2012). Meta-analytic evidence for a superordinate cognitive control network subserving diverse executive functions. Cognitive, Affective, & Behavioral Neuroscience, 12(2), 241–268. 10.3758/s13415-011-0083-5 PMC3660731 22282036

[IMAG.a.1312-b60] Nyberg, L. (2017). Functional brain imaging of episodic memory decline in ageing. Journal of Internal Medicine, 281(1), 65–74. 10.1111/joim.12533 27453565

[IMAG.a.1312-b61] Nyberg, L., Lövdén, M., Riklund, K., Lindenberger, U., & Bäckman, L. (2012). Memory aging and brain maintenance. Trends in Cognitive Sciences, 16(5), 292–305. 10.1016/j.tics.2012.04.005 22542563

[IMAG.a.1312-b62] Park, D. C., Lautenschlager, G., Hedden, T., Davidson, N. S., Smith, A. D., & Smith, P. K. (2002). Models of visuospatial and verbal memory across the adult life span. Psychology and Aging, 17(2), 299–320. 10.1037/0882-7974.17.2.299 12061414

[IMAG.a.1312-b63] Park, D. C., & Reuter-Lorenz, P. (2009). The adaptive brain: Aging and neurocognitive scaffolding. Annual Review of Psychology, 60, 173–196. 10.1146/annurev.psych.59.103006.093656 PMC3359129 19035823

[IMAG.a.1312-b64] Petrides, M. (2005). Lateral prefrontal cortex: Architectonic and functional organization. Philosophical Transactions of the Royal Society B: Biological Sciences, 360(1456), 781–795. 10.1098/rstb.2005.1631 PMC1569489 15937012

[IMAG.a.1312-b65] Power, J. D., Mitra, A., Laumann, T. O., Snyder, A. Z., Schlaggar, B. L., & Petersen, S. E. (2014). Methods to detect, characterize, and remove motion artifact in resting state fMRI. NeuroImage, 84, 320–341. 10.1016/j.neuroimage.2013.08.048 23994314 PMC3849338

[IMAG.a.1312-b66] Pruim, R. H. R., Mennes, M., van Rooij, D., Llera, A., Buitelaar, J. K., & Beckmann, C. F. (2015). ICA-AROMA: A robust ICA-based strategy for removing motion artifacts from fMRI data. NeuroImage, 112, 267–277. 10.1016/j.neuroimage.2015.02.064 25770991

[IMAG.a.1312-b67] Raud, L., Sneve, M. H., Vidal-Piñeiro, D., Sørensen, Ø., Folvik, L., Ness, H. T., Mowinckel, A. M., Grydeland, H., Walhovd, K. B., & Fjell, A. M. (2023). Hippocampal-cortical functional connectivity during memory encoding and retrieval. NeuroImage, 279, 120309. 10.1016/j.neuroimage.2023.120309 37544416

[IMAG.a.1312-b68] Ray, K., Ragland, J., MacDonald, A., Gold, J., Silverstein, S., Barch, D., & Carter, C. (2019). Dynamic reorganization of the frontal parietal network during cognitive control and episodic memory. Cognitive, Affective, & Behavioral Neuroscience, 20, 76–90. 10.3758/s13415-019-00753-9 PMC7018593 31811557

[IMAG.a.1312-b69] Rieck, J. R., Rodrigue, K. M., Boylan, M. A., & Kennedy, K. M. (2017). Age-related reduction of BOLD modulation to cognitive difficulty predicts poorer task accuracy and poorer fluid reasoning ability. NeuroImage, 147, 262–271. 10.1016/j.neuroimage.2016.12.022 27979789 PMC5303662

[IMAG.a.1312-b70] Saad, Z. S., Gotts, S. J., Murphy, K., Chen, G., Jo, H. J., Martin, A., & Cox, R. W. (2012). Trouble at rest: How correlation patterns and group differences become distorted after global signal regression. Brain Connectivity, 2(1), 25–32. 10.1089/brain.2012.0080 22432927 PMC3484684

[IMAG.a.1312-b71] Salthouse, T. A. (2009). When does age-related cognitive decline begin? Neurobiology of Aging, 30(4), 507–514. 10.1016/j.neurobiolaging.2008.09.023 19231028 PMC2683339

[IMAG.a.1312-b72] Schaefer, A., Kong, R., Gordon, E. M., Laumann, T. O., Zuo, X.-N., Holmes, A. J., Eickhoff, S. B., & Yeo, B. T. T. (2018). Local-global parcellation of the human cerebral cortex from intrinsic functional connectivity MRI. Cerebral Cortex, 28(9), 3095–3114. 10.1093/cercor/bhx179 28981612 PMC6095216

[IMAG.a.1312-b73] Selmeczy, D., & Ghetti, S. (2025). Development of episodic memory. In Encyclopedia of the human brain (pp. 236–249). Elsevier. 10.1016/B978-0-12-820480-1.00130-3

[IMAG.a.1312-b74] Sherman, L. E., Rudie, J. D., Pfeifer, J. H., Masten, C. L., McNealy, K., & Dapretto, M. (2014). Development of the default mode and central executive networks across early adolescence: A longitudinal study. Developmental Cognitive Neuroscience, 10, 148–159. 10.1016/j.dcn.2014.08.002 25282602 PMC4854607

[IMAG.a.1312-b75] Shing, Y. L., & Lindenberger, U. (2011). The development of episodic memory: Lifespan lessons. Child Development Perspectives, 5(2), 148–155. 10.1111/j.1750-8606.2011.00170.x

[IMAG.a.1312-b76] Singh-Manoux, A., Kivimaki, M., Glymour, M. M., Elbaz, A., Berr, C., Ebmeier, K. P., Ferrie, J. E., & Dugravot, A. (2012). Timing of onset of cognitive decline: Results from Whitehall II prospective cohort study. BMJ, *344*, d7622. 10.1136/bmj.d7622 PMC3281313 22223828

[IMAG.a.1312-b77] Small, S. A. (2001). Age-related memory decline: Current concepts and future directions. Archives of Neurology, 58(3), 360–364. 10.1001/archneur.58.3.360 11255438

[IMAG.a.1312-b78] Sneve, M. H., Grydeland, H., Nyberg, L., Bowles, B., Amlien, I. K., Langnes, E., Walhovd, K. B., & Fjell, A. M. (2015). Mechanisms underlying encoding of short-lived versus durable episodic memories. Journal of Neuroscience, 35(13), 5202–5212. 10.1523/JNEUROSCI.4434-14.2015 25834046 PMC6705406

[IMAG.a.1312-b79] Sørensen, Ø., Brandmaier, A. M., Macià, D., Ebmeier, K., Ghisletta, P., Kievit, R. A., Mowinckel, A. M., Walhovd, K. B., Westerhausen, R., & Fjell, A. (2021). Meta-analysis of generalized additive models in neuroimaging studies. NeuroImage, 224, 117416. 10.1016/j.neuroimage.2020.117416 33017652

[IMAG.a.1312-b80] Sowell, E. R., Peterson, B. S., Thompson, P. M., Welcome, S. E., Henkenius, A. L., & Toga, A. W. (2003). Mapping cortical change across the human life span. Nature Neuroscience, 6(3), 309–315. 10.1038/nn1008 12548289

[IMAG.a.1312-b81] Spreng, R. N., & Turner, G. R. (2019). The shifting architecture of cognition and brain function in older adulthood. Perspectives on Psychological Science, 14(4), 523–542. 10.1177/1745691619827511 31013206

[IMAG.a.1312-b82] Tromp, D., Dufour, A., Lithfous, S., Pebayle, T., & Després, O. (2015). Episodic memory in normal aging and Alzheimer disease: Insights from imaging and behavioral studies. Ageing Research Reviews, 24, 232–262. 10.1016/j.arr.2015.08.006 26318058

[IMAG.a.1312-b83] Turnbull, A., Wang, H. T., Murphy, C., Ho, N. S. P., Wang, X., Sormaz, M., Karapanagiotidis, T., Leech, R. M., Bernhardt, B., Margulies, D. S., Vatansever, D., Jefferies, E., & Smallwood, J. (2019). Left dorsolateral prefrontal cortex supports context-dependent prioritisation of off-task thought. Nature Communications, 10(1), 3816. 10.1038/s41467-019-11764-y PMC6707151 31444333

[IMAG.a.1312-b84] Turner, G. R., & Spreng, R. N. (2015). Prefrontal engagement and reduced default network suppression co-occur and are dynamically coupled in older adults: The default–executive coupling hypothesis of aging. Journal of Cognitive Neuroscience, 27(12), 2462–2476. 10.1162/jocn_a_00869 26351864

[IMAG.a.1312-b85] Vidal-Piñeiro, D., Sneve, M. H., Nyberg, L. H., Mowinckel, A. M., Sederevicius, D., Walhovd, K. B., & Fjell, A. M. (2019). Maintained frontal activity underlies high memory function over 8 years in aging. Cerebral Cortex, 29(7), 3111–3123. 10.1093/cercor/bhy177 30137326

[IMAG.a.1312-b86] Walhovd, K. B., Fjell, A. M., Sørensen, Ø., Mowinckel, A. M., Reinbold, C. S., Idland, A.-V., Watne, L. O., Franke, A., Dobricic, V., Kilpert, F., Bertram, L., & Wang, Y. (2020). Genetic risk for Alzheimer disease predicts hippocampal volume through the human lifespan. Neurology. Genetics, 6(5), e506. 10.1212/NXG.0000000000000506 33134508 PMC7577559

[IMAG.a.1312-b87] Wechsler, D. (1999). Wechsler abbreviated scale of intelligence. 10.1037/t15170-000

[IMAG.a.1312-b88] Westphal, A. J., Wang, S., & Rissman, J. (2017). Episodic memory retrieval benefits from a less modular brain network organization. Journal of Neuroscience, 37(13), 3523–3531. 10.1523/JNEUROSCI.2509-16.2017 28242796 PMC5373132

[IMAG.a.1312-b89] Xia, H., He, Q., & Chen, A. (2022). Understanding cognitive control in aging: A brain network perspective. Frontiers in Aging Neuroscience, 14, 1038756. 10.3389/fnagi.2022.1038756 36389081 PMC9659905

[IMAG.a.1312-b90] Yeo, B. T. T., Krienen, F. M., Sepulcre, J., Sabuncu, M. R., Lashkari, D., Hollinshead, M., Roffman, J. L., Smoller, J. W., Zöllei, L., Polimeni, J. R., Fischl, B., Liu, H., & Buckner, R. L. (2011). The organization of the human cerebral cortex estimated by intrinsic functional connectivity. Journal of Neurophysiology, 106(3), 1125–1165. 10.1152/jn.00338.2011 21653723 PMC3174820

[IMAG.a.1312-b91] Zhukovsky, P., Coughlan, G., Buckley, R., Grady, C., & Voineskos, A. N. (2023). Connectivity between default mode and frontoparietal networks mediates the association between global amyloid-β and episodic memory. Human Brain Mapping, 44(3), 1147–1157. 10.1002/hbm.26148 36420978 PMC9875925

